# Post-acute Transitional Journey: Caring for Orthopedic Surgery Patients in the United States

**DOI:** 10.3389/fmed.2018.00342

**Published:** 2018-12-07

**Authors:** Nicoleta Stoicea, Samarchitha Magal, January K. Kim, Michael Bai, Barbara Rogers, Sergio Daniel Bergese

**Affiliations:** ^1^Department of Anesthesiology, Wexner Medical Center, The Ohio State University, Columbus, OH, United States; ^2^College of Medicine and Life Sciences, University of Toledo, Toledo, OH, United States; ^3^College of Medicine, The Ohio State University, Columbus, OH, United States; ^4^Department of Neurological Surgery, Wexner Medical Center, The Ohio State University, Columbus, OH, United States

**Keywords:** transition of care, total joint arthroplasty, post-acute care, in-patient rehabilitation, skilled nursing facility, home health care, orthopedic trauma patients

## Abstract

As the geriatric population in the United States continues to age, there will be an increased demand for total hip and total knee arthroplasties (THAs and TKAs). Older patients tend to have more comorbidities and poorer health, and will require post-acute care (PAC) following discharge. The most utilized PAC facilities following THA and TKA are skilled nursing facilities (SNFs), in-patient rehabilitation facilities (IRFs), and home with home health care (HHC). Coordination of care between hospitals and PACs, including the complete transfer of patient information, continues to be a challenge which impacts the quality of care provided by the PACs. The increased demand of hospital resources and PACs by the geriatric population necessitates an improvement in this transition of care process. This review aims to examine the transition of care process currently utilized in the United States for orthopedic surgery patients, and discuss methods for improvement. Employing these approaches will play a key role in improving patient outcomes, decreasing preventable hospital readmissions, and reducing mortality following THA and TKA. The extensive nature of this topic and the ramification of different types of healthcare systems in different countries were the determinant factors limiting our work.

## Introduction

Epidemiology research shows that as the baby boomer generation ages, both life expectancy and old-age dependency ratio is predicted to rise alongside the incidence of osteoporosis and related fractures. The number of individuals 85 years and older are expected to triple from 2012 to 2050 (1, 2). The geriatric population is predicted to be more functionally dependent, have a greater chance of utilizing health care resources following hospital care. The anticipated increase in demand for hospital resources, including orthopedic surgical procedures, will make transition of care ever more important.

The American Geriatrics Society Health Care Systems Committee defines transitional care as “the actions carried out to ensure coordination and continuity of care for patients who are transferring between different care settings or care levels” (3). Following total joint arthroplasty (TJA), many patients are discharged to post-acute care (PAC) facilities such as skilled nursing facilities (SNFs), in-patient rehabilitation facilities (IRFs), and home with home health care (HHC) (4). Therefore, it is important to identify features of the transitional process that can be enhanced to improve patient outcomes.

**Figure 1:**
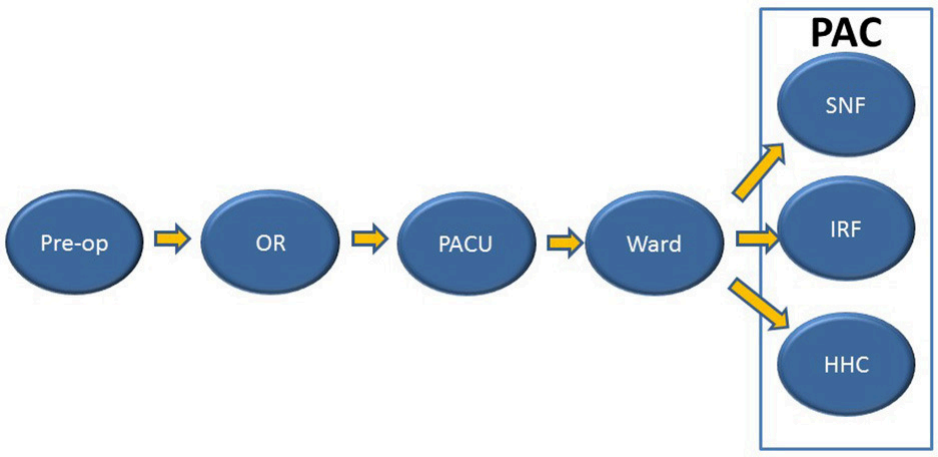
Patient flow from Pre-op to P.

We intend to identify the strengths and weaknesses of the transition of patient care (Figure 1) in total hip arthroplasty (THA) and total knee arthroplasty (TKA), while addressing methods to improve this process and, consequently, patient outcomes. Three reviewers conducted an extensive literature search accessing the following links: PubMed, Google Scholar, Science Direct, and Cochrane Review. Articles relevant to the TOC for patients undergoing TJA, specifically, THA and/or TKA are included in this review. We referenced articles published between 2010 and 2017 using the following keywords: “transition of care,” “total hip arthroplasty,” “total knee arthroplasty,” “elective surgeries,” “emergent surgeries,” “post-acute care,” “in-patient rehabilitation facility,” “skilled nursing facility,” “home health care,” “comprehensive geriatric assessment,” “linkage of care,” and “geriatric population.” Our review was conducted based on existing literature addressing the transition of care only in the United States. The extensive nature of this topic and the ramification of different types of healthcare systems in different countries were the determinant factors limiting our work. Overall, 56 articles met all the aforementioned criteria and are referenced in this review. Pertinent information regarding the TOC process of TJA patients from these studies were divided into sections by PAC types, factors, and future directions and summarized accordingly to include relevant information.

## Etiology and Demographics of Orthopedic Surgery

Over the past 20–30 years, THAs and TKAs procedures have increased significantly (5), with osteoarthritis as the primary cause of these surgeries (6). Approximately 44 million people are affected by osteoporosis in the USA; this number is expected to increase to roughly 61 million individuals by 2020 (7). Furthermore, over 50% of women older than 50 and men older than 70 years old are affected by osteoporosis (7).

Osteoporotic fractures cost the USA approximately 40 million dollars per day, with hip fractures responsible for 60% of these costs (7). Elderly age is positively associated with THA occurrence, with the greatest utilization by patients aged 70–79 years old (6). In women, the average rate of bone mineral density (BMD) loss increases with age (8), creating a greater risk of hip fracture in elderly women. Compared to outcomes associated with other osteoporotic fractures, hip fractures are associated with worse outcomes, leading to greater disability, higher costs, and increased mortality rates (8, 9). Chang and Do conducted a study investigating risk factors associated with falls in the geriatric population (Table 1) (10). Annually, one third of community-dwelling geriatrics ages 65 years and older will experience a fall, with an increasing occurrence with age; furthermore, 10–15% of these falls are severe enough to cause a fracture (8).

**Table 1:** Falls in women and men ages 65 and older—contributing factors (10).

**Medical risk factor**	**Odds ratio**
**WOMEN**	
Stroke	2.20
Urinary incontinence	1.74
Arthritis	1.67
Heart disease	1.67
Mood/anxiety disorder	1.61
GI disorder	1.56
COPD	1.51
Diabetes	1.42
Osteoporosis	1.42
Eye disorder	1.37
Hypertension	1.21
**MEN**	
Alzheimer's disease/dementia	3.06
Parkinson's disease	3.00
Stroke	2.36
Mood/Anxiety disorder	1.76
Eye Disorder	1.49
Arthritis	1.44
Urinary incontinence	1.39
Obesity	1.30
Diabetes	1.11

Hospital readmission rates increase between primary, revision, and staged revision TKAs, with primary accountable for the lowest and staged revision for the highest readmission rate (11). Patients with more comorbidities have worse outcomes following TJA, including higher incidence of complications and greater length of stay (LOS). The most common two comorbidities are diabetes mellitus and obesity, followed by cardiovascular disease (12–14). While most literature focuses on the transition of care from the hospital to outside care facilities, it is important to note that there are transitions of care within the hospital itself. Patients are moved from the operating room (OR) to the post-anesthesia care unit (PACU) immediately following surgery, then to the ward once they are considered stable. The selection of a particular PAC facility following hospital-discharge is multifactorial, involving insurance, family, and social support, and the type of multidisciplinary care required.

Most patients undergo postoperative physical therapy (PT) following TJA. Haghverdian et al. found that there is a lack of continuity of PT during the transition from hospital to SNF, which may account for the 73–50% decline in distance ambulated in SNF from Day 0 to Day 1 when compared to the last hospital session (15). Given the increasing prevalence of these surgical procedures along with PAC utilization, understanding the transition of care process is essential to improving patient outcomes.

## Pros and Cons of Post-Acute Care Settings following Orthopedic Surgery

Following THAs and TKAs, most patients are sent to one of the following PAC facilities: IRFs, SNFs, or HHC (4). From 2005 to 2008, 87% of patients on Medicare utilized PACs after undergoing TJA (16). While each PAC provides specialized care, identifying individual strengths and weaknesses among them is essential to providing appropriate care based on the severity and type of illness.

- **Inpatient rehabilitation facilities** are managed by physicians and allow for more advanced medical care (17). Patients in IRFs generally have more complex medical conditions and comorbidities, exhibit greater functional dependence, and experience increased pain (17). Although patients in IRFs are generally discharged with lower functional independence, they exhibit greater overall progress in regards to functionality, as compared to patients in SNFs (17). Additionally, IRFs are characterized by more time-efficient care compared to SNFs (17, 18). For IRFs to be Medicare certified, they must deliver at least 3 h of therapy per day for 5 days a week (16).
- **Skilled nursing facilities** provide constant care by a staff of registered nurses (RNs) and licensed practical nurses (LPNs) for patients who typically require intensive medical care (11). To individualize patient care, SNFs also utilize a multidisciplinary approach, collaborating with physical therapists, occupational therapists, speech-language pathologists, and audiologists (11). From 2005 to 2008, 37% of Medicare beneficiaries utilizing PACs following TJAs employed SNFs (16). Moreover, SNFs were found to provide more cost-efficient care than IRFs (17, 18); SNF patients generally exhibited greater functional independence than IRF patients (16). Studies controlling for patient demographics, health conditions, functional independence, comorbidities, and services demonstrated that SNFs delivered superior care over IRFs and HHC when addressing self-care capabilities; however, once accounting for the patient's LOS at the PAC, none of the aforementioned facilities exhibited a significant advantage in self-care therapy and functionality over the other (19).
- **Home health-care** offers short-term services such as wound care, education for patients and caregivers, nutritional interventions, injections, and monitoring of critical medical conditions^1^ In contrast to the services offered by other PACs, HHC provides the least amount of therapy and has a significantly longer length of stay (LOS) (Table 2) (16, 20). Patients discharged to HHC generally maintained greater functional independence, were younger, and had fewer comorbidities than those discharged to SNFs and IRFs (Table 2) (4, 16, 21). Furthermore, female patients who are older and on Medicaid or uninsured were less likely to receive HHC (22). Keepnews et al. found that 78% of patients who underwent therapy with HHC regained much functional capacity (23). Studies controlling for patient demographics, age, gender, and comorbidities concluded that patients in HHC were less likely to experience serious post-operative complications when compared with those in SNFs or IRFs, therefore ensuring better outcomes (4, 20, 24).

**Table 2:** Length of stay, comorbidity, and age by post-acute care facility (16, 19).

**Type of surgery**	**THA**			**TKA**		

**Post-acute care facility**	**Inpatient rehabilitation facility**	**Skilled nursing facility**	**Home health care**	**Inpatient rehabilitation facility**	**Skilled nursing facility**	**Home health care**
Length of stay (days)	5.31	5.36	4.30	4.49	4.76	4.31
Patients with comorbidities (%)	12.1	4.2	2.4	3.9	3.4	3.1
Mean age (years)	71.1	65.4	57.0	69.0	67.9	60.7

Medicare covered 9.6 million PAC utilizations in 2013, with a consistent increase in spending from 2001 to 2012. An increase in PAC spending since 2001 has been reported, with no simultaneous increase in post-acute quality of care. While the risk profiles of patients treated in IRFs and SNFs can be similar, Medicare spent up to 60% more on extensive programs and services offered by IRFs in 2014. The differences in Medicare payment plans amongst the PACs contribute to the varying usage of these facilities (25).

## Social Factors Associated with Post-Acute Orthopedic Care

### Race Disparities

Racial and ethnic minority groups, such as African Americans and Hispanics, have lower utilization of THA and TKA that is not fully explained by lower disease prevalence nor by disability (26). Looker et al. studied the correlation between diabetes and fracture risk in different ethnic groups and found a stronger correlation between diagnosed diabetes and fractures in non-Hispanic blacks and Mexican Americans vs. non-Hispanic whites (27). Low BMD is consistently indicative of a higher fracture risk in men 65 years and older in white, African American, Asian, Hispanic, and other ethnicities (28). Interestingly, minorities were associated with lower rates of TKA utilization with simultaneous higher rates of adverse health outcomes when undergoing such procedures, even after adjustment for patient-related and health-care system factors (29). Comorbidities, low BMD, and minority status may contribute to worse outcomes in THA and TKA patients.

### Gender Disparities

Compared to men of the same age, the lifetime fracture risk for 60-year-old women is almost doubled (44% risk for women vs. 25% risk for men) (30). Hormone levels influence the prevalence of fractures; low estrogen level is considered a significant risk factor for bone loss and osteoporosis in females (31). Furthermore, in postmenopausal women and middle-aged men, decreased sex hormones and increased iron levels account for bone loss (32). Gender also affects post-surgical destinations—females who underwent THA or TKA were more likely to be discharged to IRFs (33). Cherian et al. found that while there was no significant difference between men and women in implant survivorship and Knee Society Scores after TKA, men experienced significantly better functional outcomes (34).

### Socioeconomic Disparities

Among patients who underwent TKA, those with higher socioeconomic status, defined as insured with higher education, experienced better post-operative outcomes than those with lower socioeconomic status (35). Additionally, lower levels of education and social support were significantly associated with higher levels of pain and worse functional outcomes 6 months after TJA (Table 3) (36). According to a study analyzing post-discharge expenses over a 12-month period, 49% of TJA patients were discharged to PACs, accounting for over 70% of the total post-discharge costs (37). Compared to insured patients, uninsured patients received less care following TJA (22). Lower socioeconomic status is correlated with a higher prevalence of TJA-related adverse events.

**Table 3:** Proportion of patients with pain and functional dependence by education level (36).

**Education level**	**No college**	**Some college**	**College graduate**
Pain (%)	92.3	66.3	19.6
Functional dependence (%)	93.6	68.6	19.8

## Mental Health Associated with Post-Acute Orthopedic Care

Increased evidence declares mental health as a key determinant of overall physical health, therefore playing an important role in TJA patient outcomes. Inadequate mental health, triggered by depression, anxiety, and impaired cognitive status, is associated with poor physical health. Severe osteoarthritis of the knee is a risk factor for developing a fear of falls; avoidance of activities triggering this fear eventually leads to deterioration of physical health (38). In THA and TKA patients, preoperative “pain catastrophizing,” anxiety, depression, and impaired cognitive status are significant predictors of increased postoperative pain and worse functioning (39, 40). Conversely, social support is positively correlated to patient-perceived well-being and postoperative satisfaction after undergoing TJA, while less education and social support correlates to worse outcomes, even up to 6 months postoperatively (36). Furthermore, postsurgical pain is inversely correlated with optimism (40). Although the measured magnitude of improvement was similar in distressed and non-distressed patients, distressed patients do not return to comparable functional and psychosocial baselines (41).

Mental health factors are often overlooked. The clinical manifestation of psychological illness may not be well known to the patient or health care providers, leading to an improper diagnosis of the presenting symptoms. This could contribute to poor discharge planning and a decreased quality of psychological care provided at the PAC. Mental health disorders are much more prevalent than commonly believed. Wang et al. found that 40% of geriatric patients utilizing HHC in 2010 were diagnosed with at least one mental health disorder; however, these patients were not receiving sufficient neurological care with these HHC programs (42). There is a clear relationship between mental and physical health, so it is of utmost importance to consider this association when creating post-discharge care plans following TJA.

## New Directions for Post-Acute Orthopedic Care/Conclusion

Patients with a complicated medical history (older age, increased comorbidities, cognitive impairment, and previous admission to PACs) were identified as requiring advanced care and exhibiting potentially worse outcomes; cognitive impairment presented as the major driver for advanced care (3). Hospital readmissions are exceedingly detrimental to a patient's health. Kates et al. published retrospective data from April 2005 to September 2010, and concluded that approximately one out of every six readmissions were considered avoidable with the appropriate interventions (43). Providers should address risk factors for postdischarge adverse events prior to surgery for better patient outcomes (4). Several studies indicate that TJA rates are increasing in the United States (6), which further highlights the importance of optimizing the transition of care process.

### Comprehensive Geriatric Assessment (CGA)

Preoperative CGA should be administered to evaluate and identify at-risk patients for mortality, extended LOS, and post-discharge institutionalization in the elderly (44). According to UpToDate®, CGA is “a multidisciplinary diagnostic and treatment process that identifies medical, psychosocial, and functional limitations of a frail older person in order to develop a coordinated plan to maximize overall health with aging” (45). CGA evaluates the patient's general medical history, functional independence levels, history of falls, incontinence issues, pain levels, social support, depression history, vision or hearing complications, and durable power of attorney status (45). Early PT intervention following THA and TKA proved to significantly improve motor functioning and health-related quality of life based on CGA evaluation (46). Furthermore, it is essential to differentiate between old age and frailty. This can be achieved by using different frailty scales (Edmonton Frail Scale, Clinical Rockwood Scale) measuring cognition, functional performance, general health status, functional independence, social support, pharmacological condition, nutritional aspect, mental condition, and mental continence (47). CGA and frailty scores provide the information required to develop individual treatment pathways for geriatric patients, thereby optimizing the transition of care.

### Communication Between Care Facilities

The inadequate transfer of patient information between care facilities is a key issue in the transition of care process. The following patient information was deemed necessary to ensure smooth transfer between facilities: personal information, medical history, medication records, and post-discharge instructions (3). In 2013, the Joint Commission identified the following actions to improve transitions: strong support for the transition process by leadership, amicable relationships between care providers, a collaborative approach to care, interpersonal communication with written and electronic transfer of information, patient and family education, a continuous electronic health record (EHR), and assigned tasks for individuals involved in the transition process (48). Continuity of EHR systems between healthcare facilities aids in the transfer of patient information during transitional care; however, ensuring electronic medical records are up to date with the correct patient information is important (3). Additionally, involving pharmacists in the transitional process for medication reconciliation can have a positive impact (48). Good working relationships between the care providers of the facilities involved in transitions are often undervalued, yet necessary in the transfer of patient information. For example, a sending provider would more likely use a personal platform, as opposed to solely relying on the EHR, to communicate critical patient information to the receiving provider if they have established rapport (3). Moreover, some long-term care facilities and retirement homes require a face-to-face discussion between providers before initiation of patient transfer to prevent potential miscommunication or misinterpretation of instructions (3). Finally, the presence of family members assures smooth transitions, as they can provide the healthcare team supplementary medical history information (3). In the case of HHC, the home health staff is responsible for coordinating patient care, which includes maintaining regular communication between the patient, physician, and anyone involved in providing care to the patient ^1^. The post-operative transitional process is a multidisciplinary and collaborative effort, therefore it is important that each member of the healthcare team is held accountable for specific tasks related to transitions (48).

### Hospital-PAC Linkages

The aforementioned methods used to improve information transfer between facilities allude to the establishment of hospital-PAC linkages in the United States. The Affordable Care Act (ACA) created the Hospital Readmissions Reduction Program (HRRP), effective in 2012, focused on reducing hospital readmissions by penalizing hospitals with high readmission rates (49). The HRRP indirectly incentivizes hospitals to establish relationships with PACs, allowing for improved discharge planning and reduced readmission rates. After the implementation of the ACA, studies investigating the rates of hospital readmissions between institutions with and without SNF linkages found a correlation between well-established hospital-SNF linkage and reduced readmissions (50, 51). While most studies focused on hospital-SNF linkages, establishing relationships between hospitals and HHC providers is essential for patient care monitoring and recovery. Some programs send home health nurses to patients' homes within 2 days of their discharge; additionally, this program stresses the importance of properly managing prescribed medications, patient education, intervention plans with collective input from different departments, and consistent follow-up appointments for 1 month (48). Preliminary data showed reduced readmission rates ranging from 8.8 to 15.8 percent. This allows for regular follow-ups and reliable patient monitoring, thus effectively improving the quality of provided care.

Hospital-PAC linkages and programs fall under the umbrella of the perioperative surgical home (PSH) model applied for the first time in orthopedic surgeries. The major goal was to create a patient-centered, team-based model of care that would guide patients through the complete surgical experience. This model encompasses all aspects of care from the pre-operative optimization process through the post-discharge period. The PSH model was created in direct response to Centers for Medicare & Medicaid Services implementing a Comprehensive Care for Joint Replacement (CJR) bundled payment model, which held hospitals financially accountable for quality and cost of an entire joint replacement episode of care. By implementing the PSH model, hospitals have been able to reduce the length of stay without an increase in complications or readmissions, leading to greater reimbursements under the CJR payment plan (52). Enhanced Recover After Surgery pathway has been implemented with proven clinical benefits of reducing length of stay, cost, and perioperative complications (53).

As the population curve in this country continues to trend upwards, there is an increased risk of falls demanding emergency orthopedic interventions, leading to increased utilization of hospital resources, and prompting the improvement of the transition of care process.

Most hospital readmissions and poor outcomes can be attributed to preventable causes, pointing toward discharge planning as the principal weakness in the transition process. Utilization of PACs can be costly and, in some cases, accounts for over 1/3 of the overall cost of lower limb TJA (37). Additionally, there is cost and use variation amongst PAC facilities following TJA, with no clear relationship between type of facility used and patient outcomes (54).

Consequently, methods to improve discharge planning, such as preoperative CGA, comprehensive communication strategies between facilities, and hospital-PAC linkages, are instrumental in improving the transition process. Patient-centered initiatives focused on transition of care should be further evaluated in order to improve geriatric patient outcomes following THA and TKA through reduced hospital readmission rates, morbidity, and mortality.

## Author Contributions

All authors listed have made a substantial, direct and intellectual contribution to the manuscript, and approved it for publication. NS and SM contributed equally to the elaboration of the final version of this manuscript.

### Conflict of Interest Statement

The authors declare that the research was conducted in the absence of any commercial or financial relationships that could be construed as a potential conflict of interest.

## Footnotes

^1^*What's Home Health Care?* Medicare.gov. Available online at: https://www.medicare.gov/what-medicare-covers/home-health-care/home-health-care-what-is-it-what-to-expect.html.
